# Focus on 1,25-Dihydroxyvitamin D3 in the Peripheral Nervous System

**DOI:** 10.3389/fnins.2019.00348

**Published:** 2019-04-12

**Authors:** Pierre Antoine Faye, François Poumeaud, Federica Miressi, Anne Sophie Lia, Claire Demiot, Laurent Magy, Frédéric Favreau, Franck G. Sturtz

**Affiliations:** ^1^EA 6309, Myelin Maintenance and Peripheral Neuropathies, Faculties of Medicine and Pharmacy, University of Limoges, Limoges, France; ^2^Department of Biochemistry and Molecular Genetics, University Hospital of Limoges, Limoges, France; ^3^CHU de Limoges, Reference Center for Rare Peripheral Neuropathies, Department of Neurology, Limoges, France

**Keywords:** calcitriol, peripheral nervous system, neuronal-cell differentiation, synergistic effects, myelin process

## Abstract

In this review, we draw attention to the roles of calcitriol (1,25-dihydroxyvitamin D3) in the trophicity of the peripheral nervous system. Calcitriol has long been known to be crucial in phosphocalcium homeostasis. However, recent discoveries concerning its involvement in the immune system, anti-cancer defenses, and central nervous system development suggest a more pleiotropic role than previously thought. Several studies have highlighted the impact of calcitriol deficiency as a promoting factor of various central neurological diseases, such as multiple sclerosis, amyotrophic lateral sclerosis, Parkinson’s disease, and Alzheimer’s disease. Based on these findings and recent publications, a greater role for calcitriol may be envisioned in the peripheral nervous system. Indeed, calcitriol is involved in myelination, axonal homogeneity of peripheral nerves, and neuronal-cell differentiation. This may have useful clinical consequences, as calcitriol supplementation may be a simple means to avoid the onset and/or development of peripheral nervous-system disorders.

## Epidemiological Data and the General Function of Vitamin D3

For decades, the role of calcitriol was thought to be limited to phosphocalcium metabolism. Recent results have highlighted the role of this hormone in other functions (Garabédian, 2000; Christakos et al., 2016), which include the regulation of tissue proliferation, cell differentiation, and apoptosis, as well as regulation of the cardiovascular and immune systems. Indeed, the active form of vitamin D3 has been shown to regulate inflammation by regulating the synthesis of several cytokines and lymphocyte migration, with anti-cancer activities (Baeke et al., 2010). Based on cellular and animal models, Kalueff and Tuohimaa (2007) suggest that calcitriol has a major role in the genesis, development, and maintenance of central nervous system in adulthood. As shown in animal experiments, calcitriol may regulate rat brain development. Rats born to a mother that was vitamin D3-depleted during pregnancy were shown to have brain malformations, such as cortical atrophy associated with ventricular dilation (Eyles et al., 2005). Another study has reported the synthesis of calcitriol within the central nervous system, thus regulating its functioning and exerting neuroprotective effects (Eyles et al., 2003). Marini et al. (2010) observed that *in vitro* calcitriol delays cell proliferation and induces cell differentiation in HN9.10 embryonic hippocampal cells, with the formation of axons and dendrites. Overall, these findings suggest that vitamin D3 has activities similar to other neuroactive steroids in the central nervous system (Emmanuel et al., 2002; Melcangi and Panzica, 2009). However, the exact role of calcitriol in the peripheral nervous system is still unclear. The aim of this review was to gather available data concerning the role of calcitriol in the peripheral nervous system during its development and maintenance.

**FIGURE 1:**
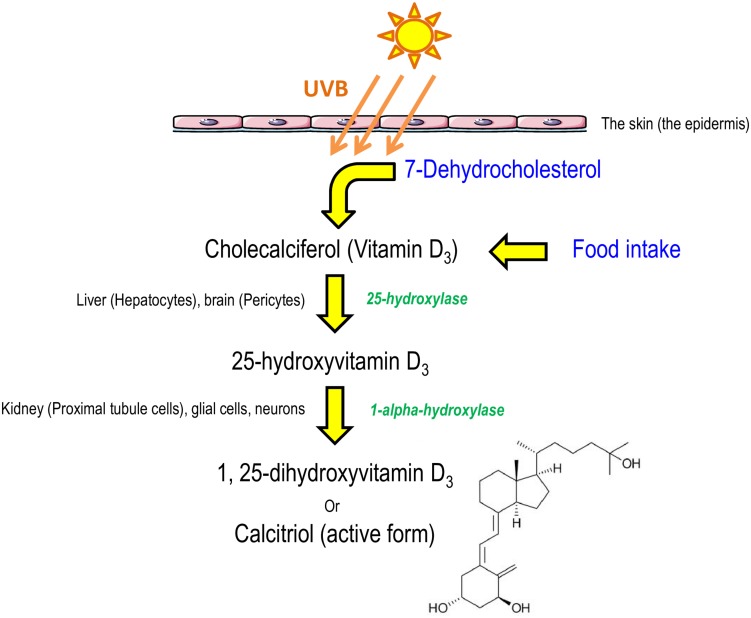
Schematic representation of calcitriol synthesis in humans. Cholecalciferol, from food intake or derived from 7-dehydrocholesterol after sun exposition, is converted to calcitriol, the active form, by two hydroxylations (Emmanuel et al., 2002; Eyles et al., 2005; El-Atifi et al., 2015).

Although all the calcitriol functions may not yet be known, the chemical characteristics have been extensively investigated. The precursor of calcitriol is vitamin D or calciferol, which is synthesized in the skin or ingested with food. This precursor is biologically inactive and subjected to double hydroxylation, first in the liver and then in the kidney, to produce the biologically active compound, 1,25-(OH)_2_-vitamin D3 or calcitriol (Figure 1). It is well known to regulate the expression of numerous target genes through the nuclear vitamin D receptor (VDR), which belongs to a common family of steroid receptors that also includes steroid, glucocorticoid, and retinoic acid receptors (Kalueff and Tuohimaa, 2007). Vitamin D deficiency is widely found worldwide (Holick, 2006). For example, the prevalence of vitamin D insufficiency was 77% in the United States population in 2004 (Ginde et al., 2009). However, reference values vary widely between countries. According to Rosen (2011) only 25-OH-vitamin D3 prohormone blood levels can accurately estimate vitamin D3 input from cutaneous synthesis and dietary intake, in contrast to 1,25-(OH)_2_-vitamin D3. The measurement of 1,25-(OH)_2_-vitamin D3 is mainly reserved for patients with kidney insufficiency. Several countries consider that serum levels of 25-OH-vitamin D3 below 10 ng/ml indicate vitamin D deficiency. Vitamin D “insufficiency” is characterized by serum levels between 10 and 30 ng/ml, an “appropriate” level between 30 and 100 ng/ml, and a “toxic” level by values above 100 ng/ml (Rosen, 2011). However, in the United States, the Endocrine Society has established different threshold levels. Vitamin D deficiency is diagnosed in patients with serum levels of 25-OH-vitamin D3 below 20 ng/ml, “sufficiency” between 30 and 40 ng/ml, and toxicity above 50 ng/ml (Ross et al., 2011). In addition, these different thresholds are those used to measure phosphocalcium homeostasis. These thresholds could be different for other functions of the nervous system and, if so, they are yet to be determined.

## Mechanistic and Molecular Interactions of Vitamin D3

Systemic action of Vitamin D3 requires a metabolization and an activation. Vitamin D3 metabolism is a multiple-step multiple-organ process which will be recalled thereafter. Once activated vitamin D3 will act upon several genes at a transcriptional level, in cooperation with other factors such as fat-soluble vitamin derivatives.

### Vitamin D3 Metabolism

Calcitriol levels are precisely regulated by the mitochondrial hydroxylases, cytochrome P450C1α (CYP27B1) and P450C24 (CYP24), which catalyze the bioactivation and degradation of vitamin D3 metabolites in most target cells (Hii and Ferrante, 2016). The blood level of calcitriol is auto-regulated through the stimulation of the CYP24 enzyme (VanAmerongen et al., 2004). In addition, calcitriol also inhibits CYP1 (renal 1 α hydroxylase involved in the second hydroxylation of vitamin D3) activity, thus forming a negative feedback loop to maintain normal levels (Issa et al., 1998). Finally, most calcitriol is excreted as calcitroic acid. The serum half-life of 1,25-(OH)_2_-vitamin D3 is approximately 4–6 h, whereas the serum half-life of 25-OH-vitamin D3 is approximately 10–21 days (Kumar, 1986). These different serum half-lives explain why 25-OH-vitamin D3 is the classical form used in serum-level measurements in humans to evaluate the body level of vitamin D3. In addition, standard protocols in the clinical lab appear to be poorly adapted to measure calcitriol levels. Indeed, liquid chromatography coupled to tandem mass spectrometry (LC-MS/MS) appears to be the most appropriate, but it is expensive and not used by most laboratories (Spanaus and von Eckardstein, 2017). This sensitive technique is used for calcitriol measurement because absolute levels of 25-OH-vitamin D3 and 1,25-(OH)_2_-vitamin D3 differ by a factor of 1000. The renal 1-alpha hydroxylation of 25-OH-vitamin D3 to 1,25-(OH)_2_-vitamin D3 is highly regulated by the serum concentration of parathyroid hormone, calcium, and phosphate. It is well known that a wide variety of extra-renal cells can produce calcitriol from 25-OH-vitamin D3 by the enzyme 1 α hydroxylase *in vitro*, including activated macrophages, keratinocytes, and cells of the central nervous system, such as neurons and microglial cells. However, the regulation of hydroxylation in these cells has not been fully explored and such production of calcitriol appears to not be finely regulated by renal production (VanAmerongen et al., 2004). Most circulating vitamin D metabolites in blood under normal physiological conditions are bound to vitamin D-binding protein or albumin and transported to a large number of target organs (VanAmerongen et al., 2004).

### Vitamin D3 and the Vitamin D Receptor (VDR)

**FIGURE 2:**
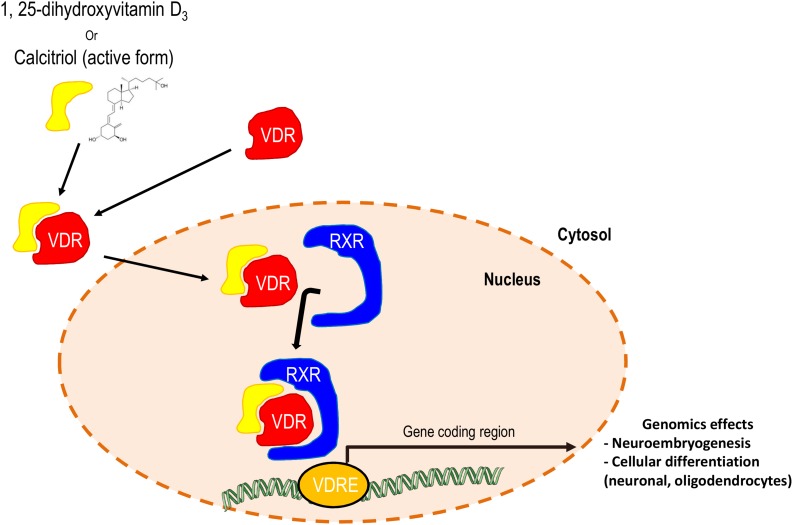
Schematic representation of the synergetic effects of calcitriol and the retinoid X receptor on the expression of genes with neuronal roles. RXR, retinoid X receptor; VDR, vitamin D_3_ receptor; VDRE, VDR responsive element.

Vitamin D is converted into its hydroxylated derivative, 1,25-(OH)_2_-vitamin D3, by two successive hydroxylations, one in the liver and one in the kidneys. Its liposolubility allows calcitriol to pass through cell membranes without a transporter. Within the cell, the vitamin D receptor (VDR), a member of the nuclear-receptor superfamily, mediates the biological activity of 1,25-(OH)_2_-vitamin D3 by regulating gene expression, similarly to other steroid hormone receptors (Figure 2). Following a conformational change, the VDR regulates gene transcription by binding to hexameric core-binding motifs in the promoter regions of target genes (Issa et al., 1998). The vitamin D-VDR endocrine system has been identified in nearly all nucleated cells. Microscopic autoradiography of the VDR has identified the target organs for vitamin D, especially the brain and spinal cord, for which there is a high binding rate (Stumpf, 2012). Although not fully understood, VDR could be involved in the development of a variety of neurological illnesses.

When entering a target cell, calcitriol dissociates from vitamin D-binding protein (the transporter of vitamin D in blood), diffuses across the plasma membrane, binds to the VDR, and the formed complex migrates to the nucleus. The activated VDR dimerizes with another nuclear receptor, the retinoic acid receptor (RXR). This RXR/VDR/calcitriol heterodimer binds to the vitamin D responsive element (VDRE), a specific sequence in the promoter region of target genes. Upon binding to the VDRE, the heterodimer activates or suppresses gene transcription. VDRs can also form homodimers but their functional significance is not known (VanAmerongen et al., 2004). In addition, efficient transcription requires co-activator or co-repressor proteins, such as Smad3, an effector of the TGF beta pathway (VanAmerongen et al., 2004). In the calcitriol pathway, Smad 3 acts as a coactivator and Smad 7 abrogates the Smad3-mediated VDR response. Cells of the central nervous system (microglia, neurons, and astrocytes) express VDR and can respond directly to calcitriol (Emmanuel et al., 2002).

Calcitriol has also been reported to modulate rapid non-genomic actions mediated through various mechanisms, such as the activation of G-protein coupled receptors and downstream protein kinase C (PKC), mitogen-activated protein kinase (MAPK) pathways, phospholipases A2 and C, and the opening of Ca^2+^ and Cl^–^ channels (Buitrago et al., 2013; Hii and Ferrante, 2016). However, these various effects have yet to be reported in cells of the nervous system.

### Vitamin D3 and Synergistic Effects With Other Vitamins

The synergistic interactions between fat-soluble vitamins have been suggested since several decades and particularly between vitamin A and vitamin E in the field of lipid peroxidation (Tesoriere et al., 1996). However, the interaction of vitamin D3 with other fat-soluble vitamins is also suggested through different mechanisms and based on different responses induced by vitamin D3 *in vitro* or *in vivo*. Indeed, vitamin D3 has been shown to regulate the growth and differentiation of a number of various cell types *in vitro*, including bone, immune and hematopoietic cells, and keratinocytes, as well as cancer cells. However, *in vivo*, these responses are achieved at toxic doses that cause severe hypercalcemia (Issa et al., 1998). These observations suggest that the effects of calcitriol underline synergistic effects between other hormones or molecules at lower concentrations.

Firstly, vitamin D3 appears to have synergistic effects with other fat-soluble vitamins, such as vitamin K, particularly for bone and cardiovascular health (van Ballegooijen et al., 2017b). Regarding bone homeostasis, in an experimental study, Kerner et al. (1989) described that osteoblast-specific expression of osteocalcin, a vitamin K-dependent protein, is controlled at the transcriptional level by the calcitriol within the promoter of the osteocalcin gene. These results were supported by Sergeev et al. (1987), in a rat model, showing that VDR can undergo gamma-carboxylation in the presence of vitamin K, which putatively interferes with its nuclear functions through VDREs. In an experimental study investigating osteoporosis in ovariectomized rats, Matsunaga et al. (1999) reported that the combined treatment with vitamin D3 and K is more effective to prevent osteoporosis. In observational studies in humans, these interactions were also pointed out. In 387 hemodialyzed patients, vitamin D3 analog users present higher concentrations of bone Gla protein (BGD) indicating the role of vitamin D3 to stimulate this vitamin K-depend proteins (Fusaro et al., 2016). In the NOREPOS study among 1318 older adults, results underlined that a combination of vitamin D3 and K supplementations at low concentrations was linked with a greater hip fracture risk compared to supplementations at high concentrations or to the group supplemented with just one vitamin at low concentrations (Finnes et al., 2016). Several clinical trials support this synergetic interaction and particularly in postmenopausal osteoporosis (van Ballegooijen et al., 2017b). For instance, in an interventional, randomized and placebo-controlled study led in 172 Japanese post-menopausal women with osteopenia and osteoporosis, results showed that only vitamin K plus vitamin D3 increased bone mineral density (Ushiroyama et al., 2002). In 78 Korean post-menopausal women over 60 years of age, vitamin K treatment associated to vitamin D and calcium increased bone mineral density (Je et al., 2011). Regarding cardiovascular health, the synergy between vitamin D3 and K was also reported. Similarly, this synergy could be linked to vitamin D3-induced stimulation of vitamin K-dependent proteins, such as matrix Gla protein (MGP), which needs gamma-glutamate carboxylation to inhibit the vascular calcification (Mayer et al., 2017). Indeed, in a rodent model, vitamin K deficiency caused by warfarin treatment, promotes arterial calcifications and this occurs earlier when high doses of vitamin D are associated (Price et al., 2000). A prospective study indicates that the combined treatment of low dose of vitamin D and a low status of vitamin K promoted systolic and diastolic blood pressures increase and hypertension after 6 years of follow up (van Ballegooijen et al., 2017a). These results were supported by another study showing that this association induces a significantly higher aortic pulse wave than in subjects with isolated vitamin D3 or vitamin K deficiency, reflecting a higher aortic resistance (Mayer et al., 2017). In addition, a randomized and double-blind trial on 42 non-dialyzed patients with chronic kidney disease showed that vitamin D3 associated with vitamin K has an additive or a synergistic effect on the decrease of intima-media thickness (Kurnatowska et al., 2015). However, synergistic effect between vitamin D3 and K may only exist at optimal concentrations. Indeed, an observational single-center cohort study showed that vitamin D3 supplementation on renal-transplanted patients with vitamin K deficiency induced increased mortality and graft failures (van Ballegooijen et al., 2019). Many more trials are currently being led as one can see on web sites for registered trials^1^,^2^.

Similarly, interactions between calcitriol and vitamin E were observed and particularly to mediate cellular antiproliferative effects. The association of low doses of calcitriol and vitamin E succinate has been reported to have additive effects on the inhibition of human prostatic cancer cells LNCaP proliferation through the stimulation of VDR expression, without adverse effect on calcemia (Yin et al., 2009). An another study led on a rat model showed that vitamin D and E deficiencies have synergistic effects on rickets development (Sergeev et al., 1987). However, the additive or synergistic mechanism of this association is still unclear and requires further study.

In addition, a synergistic effect of vitamin D3 and A, which is a retinoic acid precursor, has been reported in various cellular models (breast, prostate, colon, and leukemia) but also in mycobacteria (Guilland, 2011; Greenstein et al., 2012). These effects could be linked to the dimerization between the VDR and RXR, which creates an interconnection between the calcitriol and retinoic acid cellular pathways. Indeed, retinoic acid could modulate the vitamin D3 effects. Several studies pointed out an antagonism or additive/synergetic effects between both vitamins. For instance, Kane et al. (1996) showed an inhibition by retinoic acid of the antiproliferative effect of calcitriol on colon cancer cells. However, several studies reported a synergistic effect. *In vitro*, on human prostatic cancer cells LNCaP, Blutt et al. (1997) suggested that calcitriol and retinoic acid act synergistically to inhibit the growth of cancer cells and cause accumulation of cells in G1. Carlberg et al. (1993) showed that in drosophila SL-3 cells transfected with mouse VDR or RXR genes, the VDRE was synergistically activated by RXR and VDR, but only in the presence of both factors. Regarding the nervous system, the RXR has been shown to be involved in the differentiation of oligodendrocyte progenitors into mature oligodendrocytes (de la Fuente et al., 2015), and also in neuronal differentiation (Mounier et al., 2015). It is well known that retinoic acid plays a major role during the embryological development of the central nervous system, leading the neuroectoderm to caudalize itself. On the other hand, calcitriol also plays a role in neuro-embryogenesis (Shirazi et al., 2015). Thus, it is conceivable that a synergistic interconnection between retinoic acid and calcitriol exists during nervous system development. All of the interactions between vitamin D and other fat-soluble vitamins presented above show that this field is quite large and matter for further explorations in the nervous system.

### Cardiovascular Effects and Systemic Interactions of Vitamin D3

Vitamin D3 has been suspected to play a role in cardioprotection. Indeed, VDR-deficient mice showed adverse cardiac remodeling and hypertension (Meems et al., 2011). However, in an observational, prospective and population-based cohort study, calcitriol or calcidiol plasmatic levels have failed in predicting higher risk of heart failure (Meems et al., 2016). Thus, further studies are required to investigate strong evidence-based relationship between Vitamin D3 and heart failure. On the other hand, 1,25-(OH)_2_-vitamin D3 may also induce adverse effects in humans. Another observational, prospective and population-based cohort study demonstrated that plasma calcitriol levels are associated with an elevated risk of hypertension (van Ballegooijen et al., 2015). Intriguingly and unexpectedly, cholecalciferol plasma levels are inversely associated with hypertension. However, calcitriol supplementation was shown to cause renal calcification in an experimental laboratory study led on a suckling rat model (Dostal et al., 1984), which is confirmed by the fact that, in humans, cholecalciferol supplementation is associated with kidney-stone formation, linked to increased hypercalciuria (Letavernier and Daudon, 2018).

## Roles of Vitamin D3 in the Nervous System

As reported above and in Table 1, data suggest that calcitriol has a role in the nervous system and that vitamin D3 acts as a neurosteroid (Emmanuel et al., 2002; Melcangi and Panzica, 2009). However, the role, if any, of the calcitriol in the peripheral nervous system needs to be more precisely defined.

**TABLE 1:** Different experimental models or human cohorts aimed to investigate the positive or negative role of Vitamin D3.

Level	Model	Sample size (*N* = x)	Study design	Variable	Outcome	References
Cellular	Astrocytes			Vitamin D3 supplementation	↑ GDNF, NT-3, NT-4	Neveu et al.,1994
	Primary cortical neurons (E18 rats)			Vitamin D3 + progesterone supplementation	Better neuroprotection (vs. progesterone alone)	Atif et al.,2009
	Embryonic hippocampal cells			Vitamin D3 supplementation	Delay proliferation Enhance neuronal differentiation	Marini et al.,2010
	Human renal carcinoma cells			Vitamin D3 supplementation	↑ Cell proliferation ↓ Apoptosis	Dormoy et al.,2012
	H9c2 rat embryonic myocardium cells			Vitamin D3 supplementation	Cardiac differentiation	Hlaing et al.,2014
	Follicle dermal papilla cells			VDR (KO)	Inhibition of proliferation and differentiation	Lim et al.,2014
	Human melanocytes			H2O2 + calcipotriol	↑ MFN2	Gong et al.,2015
	Rat primary Schwann cells			Vitamin D3 supplementation	↑ Synthesis of IGF-1, MBP	Sakai et al.,2015
	Pancreatic cell line INS-1 (rats)			Vitamin D3 supplementation	↑ GDAP1	Pepaj et al.,2015
	Glial cells			Vitamin D3 supplementation	↑ Synthesis of estrogen	Caccamo et al.,2018
Animal	Rats	112		Vitamin D3 supplementation	↓ Hippocampic iNOS synthesis	Garcion et al.,1998
		24		Vitamin D3 in utero deficiency	Cortical atrophy, Ventricular dilation, ↑ NGF and GDNF	Eyles et al.,2003
		26		Vitamin D3 supplementation + peripheral nerve trauma	↑ Axonogenesis and axon diameter	Chabas et al.,2008
		36		Vitamin D3 supplementation + Peripheral nerve trauma	↑ Neurite myelination Electro-clinical recovery	Chabas et al.,2013
		30		Vitamin D3 pre and post natal deficiency	↑ Synapses number cortical atrophy	Al-harbi et al.,2017
	Mice	15		VDR (KO)	Heterogeneity in axonal diameters and axonal partitioning (Sciatic nerve)	Sakai et al.,2015
Human	Parkinson’s disease	114	Experimental (randomized, double-blind, placebo-controlled)	Vitamin D3 supplementation	Stabilization of symptoms severity (CT or TT genotypes)	Suzuki et al.,2013
		51	Experimental (pilot randomized, double-blind, placebo-controlled)	Vitamin D3 supplementation	↑ Balance (52–66 y.o)	Hiller et al.,2018
	Alzheimer’s disease	43	Experimental (pre-post pilot, multivariate analyses)	Memantine + Vitamin D3	↑ Cognitive performance	Annweiler et al.,2012
	Multiple sclerosis	348	Experimental (phase II, multicenter, randomized, double-blind, placebo-controlled)	Vitamin D3 supplementation	Anti-inflammatory phenotype	Smolders et al.,2010
	Diabetic neuropathy	112	Experimental (non-randomized, double-blind, placebo-controlled)	Vitamin D3 supplementation	↓ Hyperesthesia and burning sensation	Shehab et al.,2015
						

*y.o., years old.*

### Vitamin D3 and Cell Differentiation

We further investigate the role of calcitriol in nervous system development, particularly neuronal cell differentiation, by focusing on the various actors known to be regulated by calcitriol, such as the Wnt signaling pathway, Sonic hedgehog (Shh), and Klotho, as well as on the putative role of progesterone to stimulate the effect of calcitriol in differentiation.

#### Wnt Proteins

Wnt proteins are cysteine-rich glycosylated proteins that control multiple processes involving neuronal development, angiogenesis, immunity, tumorigenesis, fibrosis, and stem-cell proliferation (Maiese, 2015). Wnt is also involved in nervous system development, particularly as a positive regulator of the myelination process, by promoting myelin gene expression. Tawk et al. (2011) demonstrated that the inactivation of Wnt components *in vitro* in mouse Schwann cells leads to severe dysmyelination and the inhibition of myelin gene expression. Calcitriol has been shown to disrupt Wnt/β-catenin signaling through multiple mechanisms. Hlaing et al. (2014) reported that vitamin D promotes cardiac differentiation through the negative modulation of the canonical Wnt signaling pathway and upregulation of the expression of Wnt11, *in vitro* culture of H9c2 rat embryonic myocardium cells. Lim et al. (2014) found that decreased expression of the VDR is associated with decreased expression of Wnt/β-catenin signals in follicle dermal papilla cells, inhibiting the proliferation, and differentiation of hair follicles and epidermal cells.

#### The Shh Pathway

Sonic hedgehog signaling is involved in the induction of neuronal populations in the central and peripheral nervous systems and neural stem-cell proliferation (Choudhry et al., 2014). In a recent study in an embryonic carcinoma mice cell line (P19EC), Vuong et al. (2017) clearly showed that Shh signaling regulates neuronal differentiation and neurite growth. In an experimental study using VDR-deficient mice, Teichert et al. (2011) reported that VDR-null animals overexpress Shh in keratinocytes and that such overexpression is downregulated by 1,25-(OH)_2_-vitamin D3. These results were supported by Dormoy et al. (2012) who showed that vitamin D decreases cell proliferation and increases cell death by inhibiting the Shh pathway in human renal carcinoma cells. Although the Wnt/β-catenin pathway and Shh signaling are well known to regulate the progression of spinal-cord progenitors and promote neurogenesis, particularly spinal motor-neuron development, the role of vitamin D in motor-neuron cell differentiation needs to be investigated. Further studies are necessary to clearly elucidate the role of vitamin D in neuronal cell differentiation through this pathway (Appel and Eisen, 2003; Andersson et al., 2013).

#### The Klotho Pathway

Several studies have reported a complex interaction between calcitriol activity and the Klotho gene. The Klotho gene was discovered in 1997 when mice in which this gene was silenced developed pre-mature aging syndrome (Kuro-o et al., 1997). It is highly expressed in the brain and, to a lesser extent, in other organs (Kuro-o et al., 1997). The choroid plexus is a site of abundant Klotho expression. It is well known that several factors, including phosphate and vitamin D, can regulate the production of Klotho, as well as fibroblast growth factor 23 (FGF23). Kalueff and Tuohimaa (2007) suggested that Klotho expression is upregulated by calcitriol in a murine model. FGF23 was identified as a phosphaturic hormone which is produced in the bone and controls mineral homeostasis by the regulation of calcitriol (White et al., 2000). FGF23 is known to suppress vitamin D hormone production in the kidney by downregulating renal 1α hydroxylase expression, thereby suppressing the production of calcitriol (Erben, 2016). However, little is known about the functional role of Klotho and FGF23 in the central nervous system. Although Anour et al. (2012) reported that Klotho/VDR complex mutant mice do not show obvious behavioral abnormalities, mice with a non-functioning vitamin D receptor fully restored the premature aging phenotype in Klotho deficient mice. These mice produce excessive amounts of calcitriol due to the lack of the suppressive effect of FGF23 on 1α hydroxylase expression. Thus, the premature aging phenotype in Klotho deficient mice could be caused by intoxication with the vitamin D hormone, leading to severe hypercalcemia and hyperphosphatemia and subsequent organ damage (Erben, 2016). Anamizu et al. (2005) reported that Klotho insufficiency causes atrophy and dysfunction of spinal large anterior horn cells in a mouse model deficient for Klotho, suggesting its putative role in neuronal-cell differentiation, potentially promoted by vitamin D.

#### Progesterone

Marini et al. (2010) reported that vitamin D delays cell proliferation and induces cell differentiation, with modification of soma lengthening and the formation of axons and dendrites in a study using embryonic hippocampal cells. Various observations have also shown that progesterone treatment may be beneficial in several brain-injury models (Sayeed and Stein, 2009). Although progesterone treatment of animals submitted to traumatic brain injury was shown ineffective, treatment with this steroid was effective if calcitriol was simultaneously given (Cekic et al., 2009). In addition, results show that progesterone combined with vitamin D promotes better neuroprotection against excitotoxicity than progesterone alone in an E18 rat primary cortical neurons pretreated with various concentrations of progesterone and vitamin D separately or in combination for 24 h (Atif et al., 2009). Moreover, given the role of progesterone in myelin formation in the peripheral nervous system, it could be informative to further study whether calcitriol can synergize with progesterone activity in the myelination process in the peripheral nervous system (Zárate et al., 2017). Finally, calcitriol has been shown to increase local estrogen production in glial cells through the upregulation of the aromatase enzyme (Caccamo et al., 2018). Given the role of estrogens on neuroprotection and neuronal DNA repair enzymes in rodents (Zárate et al., 2017), we suggest that calcitriol can exert a neuroprotective effect through the estrogen pathway.

#### Neuronal Cell Differentiation

Calcitriol could be used to potentiate neuronal-cell differentiation in progenitor cell lines. Indeed, Agholme et al. (2010) reported that *in vitro* pre-treatment of SH-SY5Y cells, human neuroblastoma cells, with retinoic acid, followed by culturing on an extracellular matrix in combination with a cocktail of neurotrophic factors associated with vitamin D3 treatment, generated sustainable cells with an unambiguous resemblance to adult neurons. Preliminary experiments conducted in our lab on neuronal cells with various concentrations of calcitriol suggest that calcitriol can induce motor-neuron differentiation but without any effect on proliferation. Confirmatory studies are under way.

### Axonal Homogeneity

As mentioned, vitamin D3 and its metabolites also play a role in neurites integrity. The VDR KO mouse model described by Sakai et al. (2015) underlined the involvement of calcitriol and the VDR in axonal homogeneity, integrity, and maintenance of neuromuscular junctions. Indeed, the analysis of transversal sections of sciatic nerves from VDR-deficient mice showed heterogeneity of the axonal diameters and axonal repartitioning among the nerves (Sakai et al., 2015). In addition, they showed in a rat primary Schwann cells model, that calcitriol upregulates the expression of IGF-1, a myelin basic protein which is a myotrophic and neurotrophic factor. Gao et al. (1999) showed that IGF-I deficient mice exhibit reduced peripheral nerve conduction velocities and smaller axonal diameters. They also demonstrated that IGF-1 plays a key role in the growth and development of the peripheral nervous system and that systemic IGF-1 treatment can enhance nerve function in these adults deficient mice (Gao et al., 1999).

### Anti-oxidative Activity

Anti-oxidative stress activity has been reported for calcitriol in the central nervous system (Garcion et al., 1998). Injections of lipopolysaccharide were performed *in vivo* in rat hippocampus to induce the synthesis of induced nitric oxide synthase (iNOS), which is partially involved in oxidative stress in the brain and vasodilation through nitrogen monoxide (NO) generation. This study showed significant inhibition of iNOS synthesis in the group with calcitriol treatment, suggesting a putative role of calcitriol against oxidative stress and vasodilation in the brain. Furthermore, the authors also showed that vitamin D increased the intracellular levels of glutathione, the major intracellular redox buffer, in primary cultures of newborn-rat astrocytes (Garcion et al., 1999). Although oxidative stress and inflammatory processes appear to promote calcium dysregulation with age, several endogenous steroid hormones, including vitamin D, estrogen, and insulin may counteract, at least partially, these effects (Frazier et al., 2017).

### Renin-Angiotensin System and Vitamin D

Several studies have shown an interaction between the renin-angiotensin system (RAS) and calcitriol regulation. Rammos et al. (2008) showed that vitamin D downregulates renin and vitamin D deficiency upregulates the RAS in a murine model. These results have been supported by several studies showing that renin expression and plasma angiotensin II production are elevated in VDR-null mice, leading to hypertension and cardiac hypertrophy, whereas 1,25-(OH)_2_-vitamin D3 treatment suppresses renin expression (Li et al., 2002; Yang et al., 2018). In addition, 1,25-(OH)_2_-vitamin D3 administration corrects hypertension induced by activation of the RAS in a model of 1-alpha-hydroxylase-deficient mice (Zhang et al., 2015). These renal abnormalities were also observed in a rat model of diabetes in which calcitriol blocks RAS activation (Deng et al., 2016). These interactions have also been observed in humans. In a large cohort, Tomaschitz et al. (2010) reported that serum 1,25-(OH)_2_-vitamin D3 concentrations were inversely correlated with plasma renin activity and angiotensin II levels. Calcitriol can also regulate the RAS in organs other than the kidney and perhaps in peripheral nerves, where angiotensin receptors have already been described. Indeed, Bessaguet et al. (2017) in a recent study showed that candesartan, a blocker of AT1 and AT2 receptors, prevents this type of neuropathy by acting on the RAS, in mice exhibiting sensory small fiber injury induced by resiniferatoxin treatment. They concluded that the AT2R may have neuroprotective effects (Bessaguet et al., 2017). Given the previous observation in kidney, the role of vitamin D in this pathway needs to be investigated to clarify its role in the regulation of the RAS, particularly its interaction with oxidative stress, well known to interact with the RAS (Luo et al., 2015). RAS hyperactivity associated with progression to renal damage and the modulation of calcitriol production is found in chronic kidney diseases (Santos et al., 2012).

Relationships between Vitamin D3, both cholecalciferol and calcitriol, and renal function have been extensively studied. First, renal injuries induce a decline in the glomerular filtration rate (eGFR), often associated with a reduction of 1-alpha-hydroxylase enzyme activity in kidney, inducing a decrease of plasma 1,25-(OH)_2_-vitamin D3 levels. Such low levels in the blood result in several downstream effects, such as secondary hyperparathyroidism and the modification of bone homeostasis, requiring treatment with 1,25-(OH)_2_-vitamin D3 or one of its analogs in human patients with chronic kidney diseases (Bhan, 2014). As shown by a cross-sectional study integrating results of 5 cohort studies and clinical trials, it seems that low eGFR is also associated with important decrease in Vitamin D3 catabolism (de Boer et al., 2014). Second, Vitamin D deficiency impacts in a different manner the general population and renal transplanted patients. Indeed, as shown by a prospective population-based cohort study, it seems that low calcitriol and low cholecalciferol plasma levels are not associated with decreased eGFR in the general population (Keyzer et al., 2015a). On the contrary, a prospective observational single-center cohort study in stable renal transplanted patients, showed that low 25-OH-vitamin D3 (<12 ng/mL) is associated with a rapid decline in eGFR (Keyzer et al., 2015b). Interestingly, it seems that vitamin D3 might be not “useful” to normal persons but might have an important positive effect in kidney transplanted persons. This might also be the case for people with peripheral neuropathies.

## Vitamin D3 in Neurological Disorders

It is commonly accepted that a large proportion of the population in developed countries exhibit insufficient 25-OH-vitamin D3 concentrations in the blood (Singh and Bonham, 2014). Low levels of 25-OH-vitamin D3 are associated with an increased risk of all-cause mortality (Gröber et al., 2015). Although the major sites of action of calcitriol in calcium homeostasis are the bones, kidneys, intestine, and parathyroid gland (Issa et al., 1998), the nervous system may also be involved, particularly in myelinating areas. Various associations have been reported between vitamin D status and brain diseases, such as epilepsy. 25-OH-vitamin D3 supplementation results in improved seizure control in patients with pharmaco-resistant epilepsy (Holló et al., 2012; Miratashi et al., 2017). In 2013, Zhao et al. reported a correlation between 25-OH-vitamin D3 deficiency and the prevalence of Alzheimer’s and Parkinson’s diseases (Oudshoorn et al., 2008; Zhao et al., 2013). In addition, a study in the United States reported a higher prevalence of dementia among participants with 25-OH-vitamin D3 deficiency (Buell et al., 2010). Kalueff and Tuohimaa (2007) reported the importance of vitamin D/VDR bioactivation in brain neurons, glial cells, brain macrophages, the spinal cord, and the peripheral nervous system, with putative autocrine or paracrine activity.

### Brain and Central Nervous-System Disorders

In the nervous system, vitamin D is involved in calcium trafficking, the redox status, and induction of the synthesis of synaptic structural proteins, neurotrophic factors, and deficient neurotransmitters (Mpandzou et al., 2016). Several results underline the impact of 25-OH-vitamin D3 deficiency as a promoting factor in various neurodegenerative diseases, such as amyotrophic lateral sclerosis and Parkinson’s and Alzheimer’s diseases (Evatt, 2010; Knekt et al., 2010; Mpandzou et al., 2016). The role of calcium in neurodegenerative disorders has been further studied over the last several years (Frazier et al., 2017). In humans, vitamin D deficiency has long been known to be accompanied by irritability, anxiety, depression, psychoses, and defects in mental development (Kalueff and Tuohimaa, 2007). Calcitriol deficiency is also associated with poor cognitive function in human adults, as well as in children, and could also affect brain development (Wilkins et al., 2006; Lee et al., 2009; Llewellyn et al., 2011). Eyles et al. (2003) demonstrated that rats born to vitamin D3-deficient mothers had profound alterations of the brain at birth. Changes in brain structure and a reduction in brain content of nerve-growth factor (NGF) and glial cell-derived neurotrophic factor (GDNF) suggest that low maternal vitamin D3 levels affect the developing brain (Eyles et al., 2003). These results were supported by an experimental study in a rat model with a combined prenatal and postnatal vitamin D3-deficiency (Al-harbi et al., 2017). Al-harbi et al. (2017) reported that this deficiency promoted a decrease in the number of synapses in the molecular layer of the hippocampus, associated with a reduction of cortical thickness.

Astrocytes, which are VDR expressing cells, are important immune cells and contribute to inflammation during neurological disorders. Jiao et al. (2017) reported that lipopolysaccharide-stimulated neuroinflammation in astrocytes could enhance the expression of the VDR and Cyp27B1. In contrast, vitamin D suppressed the expression of proinflammatory cytokines, such as tumor necrosis factor-α, interleukin-1β, and TLR4 *in vivo*. These results support a function of reactive astrocytes in stimulating the inflammatory response in neurodegeneration and brain injury and a putative role of vitamin D (Jiao et al., 2017).

Mascarenhas et al. reported an association between severe hypovitaminosis D and persistent, non-specific musculoskeletal pain in humans (Plotnikoff and Quigley, 2003; Mascarenhas and Mobarhan, 2004). Serum vitamin D levels have been inversely correlated with painful manifestations and associated with neuromuscular disorders, which can lead to increased pain sensitivity. Thus, vitamin D3 may also be involved in nociceptive sensitivity (de Oliveira et al., 2017). 1,25-(OH)_2_-vitamin D3 may also upregulate the expression of neurotrophic factors, such as GDNF in C6 glioma cells (Naveilhan et al., 1996), NT-3, or NT-4 in rat astrocytes (Neveu et al., 1994), TGFβ in neuroblastoma cells (Veenstra et al., 1997), and NGF in the central (Brown et al., 2003; Gezen-Ak et al., 2011) and peripheral nervous systems (Cornet et al., 1998).

Interventional studies of vitamin D3 supplementation for various central nervous system (CNS) diseases have shown promising results. In a randomized double-blind placebo-controlled trial in patients with Parkinson FokI CT and TT genotypes, 12 months of 1,200 UI/day vitamin D3 supplementation resulted in stabilization of the severity (Suzuki et al., 2013). Another randomized double-blind controlled study that assessed 4 months of vitamin D3 supplementation (10.000 UI/day) also showed improvements in balance only in 52- to 66-year-old patients with Parkinson’s disease (Hiller et al., 2018). In a single-center trial in patients with Alzheimer’s disease, co-administration of memantine with vitamin D3 (400–1000 UI/day or 100,000–200,000 UI/month) for 6 months resulted in a significant and synergistic effect on global cognitive performance (Annweiler et al., 2012). A similar protocol with memantine and vitamin D3 (100,000 UI/month) for 6 months is currently being tested in a single-center double-blind randomized placebo-controlled superiority trial to study its impact on the cognitive performance of patients with Alzheimer’s disease and similar disorders (Annweiler et al., 2011). Finally, in an observational retrospective study, 2,000 UI/day vitamin D3 supplementation for 9 months showed no significant adverse events and appeared to have beneficial effects for patients with amyotrophic lateral sclerosis. However, given the low number of patients included (37), further studies are necessary (Karam et al., 2013).

### Demyelinating Diseases

In multiple sclerosis, a demyelinating disease of the central nervous system, environmental factors may contribute to the onset of the disease, in addition to a genetic component. Poor exposure to sun light, resulting in reduced production of vitamin D3 in the skin, is thought to be a risk factor for multiple sclerosis. An association of vitamin D levels with multiple sclerosis was determined in a case control study, which showed an inverse relationship between serum 25-OH-vitamin D3 levels and the prevalence of multiple sclerosis (Pandit et al., 2013). Moreover, low 25-OH-vitamin D3 levels at birth could increase the risk of developing multiple sclerosis, as shown in a recent case-control study (Munger et al., 2016). In addition, vitamin D3 supplementation is increasingly recommended to patients with multiple sclerosis (Nystad et al., 2014). Interventional studies have also been conducted on patients with multiple sclerosis to study the impact of vitamin D3 supplementation. In an interventional single group trial, high doses of vitamin D3 (20,000 UI/day) given to patients with relapsing remitting multiple sclerosis for 12 weeks showed a shift from a pro-inflammatory to an anti-inflammatory profile (higher proportion of IL-10^+^ CD4^+^ and fewer TH1/TH2 cells) without hypercalcemia or hypercalciuria (Smolders et al., 2010). A phase I/II dose-escalation trial studying the safety of high-dose vitamin D3 supplementation (40,000 UI/day for 28 weeks, then 10,000 UI/day for 12 weeks and no supplementation for 12 weeks), associated with calcium supplementation (1,200 mg/day for 42 weeks), confirmed no significant adverse events (Burton et al., 2010). Other interventional studies for vitamin D3 supplementation in patients with multiple sclerosis are currently ongoing (Smolders et al., 2011; Dörr et al., 2012; Bhargava et al., 2014).

In a rodent model of experimental autoimmune encephalomyelitis (EAE), animals immunized against central nervous system proteins, such as myelin-basic protein, develop a paralytic disease that mimics multiple sclerosis. High doses of calcitriol have been shown to prolong survival and improve demyelination scores in the central nervous system relative to those of untreated rodents (Issa et al., 1998; Shirazi et al., 2017). Sakai et al. (2015) showed that calcitriol is essential for the synthesis of myelin basic protein, which is a main component of myelin. Indeed, rat primary Schwann cells treated with calcitriol showed increased production of myelin basic protein, suggesting calcitriol involvement in protein remyelination. Moreover, the effect of high doses of calcitriol on remyelination was investigated in C57B1/6 mice, previously treated with cuprizone, which induces oligodendrocyte apoptosis and subsequent myelin disruption. Calcitriol was able to promote the regenerative process by stimulating oligodendrocyte maturation and astrocyte activation, with a significant increase in myelination (Nystad et al., 2014). Both studies suggest an active role of calcitriol in myelination in the central and peripheral nervous systems.

### Peripheral Neuropathies

Calcitriol coordinates the biosynthesis of neurotransmitters in the central nervous system, which regulate cardiovascular autonomic function and may explain its putative role in the development of cardiovascular autonomic neuropathy (Dimova et al., 2017). In addition, Chabas et al. (2008) showed that vitamin D2 (ergocalciferol: a compound produced by yeast with effects similar to those of vitamin D3) has positive effects *in vivo* at a dose of 100 IU/kg/day in a rat model of peripheral nerve trauma. At the end of treatment, they observed a significant increase in axonogenesis and axon diameter, improving the response of sensory neurons (Chabas et al., 2008). In 2013, the same authors showed that vitamin D3 is beneficial at a dose of 500 IU/kg/day in a rat model of peripheral nerve trauma, inducing significant locomotor and electrophysiological recovery. The authors also demonstrated that 25-OH-vitamin D3 increases the number of preserved or newly formed axons in the proximal end, the mean axon diameter in the distal end, and neurite myelination in both the distal and proximal ends (Chabas et al., 2013). In an observational prospective open case-control study with 70 patients undergoing paclitaxel chemotherapy, Grim et al. (2017) reported that estimated vitamin D levels in the group without chemotherapy-induced peripheral neuropathy (CIPN) were 38.2 (24.95, 47.63) nmol/L, whereas it was 25.6 (19.7, 32.55) nmol/L in the group with CIPN. Numerous reports have linked vitamin D deficiency to an increased risk of diabetes mellitus and complications, such as neuropathy (Putz et al., 2013). Indeed, in a prospective clinical cohort study of 69 diabetic patients, Celikbilek et al. (2015) reported, that serum vitamin D levels were significantly lower in patients with diabetic peripheral neuropathy than in those without. These results were supported by an observational study showing that 25-OH-vitamin D3 levels were significantly lower in the neuropathy patient group of a 96-patient cohort with type 1 diabetes (Ozuguz et al., 2016). In addition, in a case-control study, Alamdari et al. (2015) reported that lower levels of circulating 25-OH-vitamin D3 may contribute to the risk of large-fiber neuropathy in type 2 diabetic subjects, even after adjustment for demographic variables, comorbidities, and diabetes treatment. They suggested that each 1 ng/mL increase in the concentration of seric 25-OH-vitamin D3 correlates with a 2.2 and 3.4% decrease in the presence and severity of nerve conduction velocity (NCV) impairment, respectively (Alamdari et al., 2015). Putz et al. (2014) suggested that vitamin D supplementation could have beneficial effects on neuropathic pain and may block the progression of neuronal degeneration. These authors also suggested that vitamin D deficiency could promote diabetic plantar ulcers (Putz et al., 2014). In a prospective placebo-controlled trial that included 112 type 2 diabetic patients with diabetic peripheral neuropathy and vitamin D deficiency, Shehab et al. (2015) showed that short-term oral vitamin D supplementation (50,000 UI/week for 8 weeks) improved hyperesthesia and the burning sensation, assessed by the neuropathy symptom score (NSS). However, this supplementation had no effect on the neuropathy disability score (NDS) nor nerve conduction study (NCS) (Shehab et al., 2015).

Diabetic neuropathy is associated with decreased NGF expression in human diabetic nerves (Anand et al., 1996) and vitamin D3 is also known to induce NGF synthesis in human cell lines (Fukuoka et al., 2001; Shehab et al., 2015). Thus, the observed improvement in diabetic neuropathy may be mediated through the upregulation of NGF. Recently, in an experimental randomized clinical trial on 81 women suffering from diabetic neuropathy, Nadi et al. (2017) showed that exercise combined with vitamin D supplementation decreases the complications of diabetic neuropathy. In addition, a case-report study with one Type-1 patient suffering from diabetic neuropathy, mentioned an improvement after correction of his vitamin D3 deficiency following supplementation of 50,000 UI/week for 4 weeks (Bell, 2012). On the other hand, an interventional randomized double-blind placebo-controlled trial in non-vitamin D-deficient patients with Type 2 diabetes, showed that vitamin D3 supplementation of 50,000 UI/week for 6 months provided no improvement of diabetic neuropathy (Westra et al., 2016). Placebo-controlled multi-centric studies are required to assess the role of vitamin D3 supplementation on diabetic neuropathies (Valensi et al., 2005). As previously reported, the number of studies that have investigated the role of vitamin D in the treatment of neuropathies is still limited, mostly to diabetic neuropathy.

### Charcot-Marie-Tooth Disease

Charcot-Marie-Tooth (CMT) disease is the most common form of hereditary motor and sensory neuropathy. Caused by either axonal or demyelinating alterations. More than 90 mutated genes are involved in the development of this neuropathic disease. The observed phenotype is variable but often consists of a progressive distal motor deficiency, foot deformities, or muscular atrophy (Vallat et al., 2007).

Mutations of the ganglioside-induced differentiation-associated protein 1 (*GDAP1*) gene cause autosomal dominant and autosomal recessive CMT diseases, with more than 40 different pathogenic mutations. Pepaj et al. (2015) used a proteomic approach to show that 1,25-dihydroxyvitamin D3 treatment induces overexpression of GDAP1 in a rat pancreatic beta-1 cell line. Thus, 1,25-vitamin D3 could potentially play a role in CMT, through the up-regulation of the *GDAP1* gene. Further studies are required to assess the impact of 1,25-dihydroxyvitamin D3 supplementation on the expression of the *GDAP1* gene in CMT patients and its clinical impact.

Moreover, another form of CMT disease, type 2A, is caused by mutations in the mitofusin-2 (*MFN2*) gene, which is physiologically involved in the fusion/fission of mitochondria. Preclinical studies conducted on neurons from a CMT2A mouse model showed that an MFN2 agonist could normalize mitochondrial trafficking and mobility along axons (Gezen-Ak et al., 2011). Furthermore, Gong et al. (2015) showed that treatment of human melanocytes with 0.05% H_2_O_2_ and calcipotriol (which is a structural analog of calcitriol) at doses varying from 20 to 80 nM upregulated the expression of MFN2. Thus, calcitriol could be an promising candidate in further studies on CMT2A disease.

Calcitriol has been reported to exhibit gene-dependent synergistic or antagonistic effects when co-administered with inhibitors of histone deacetylase (HDAC) (Malinen et al., 2008; Seuter et al., 2013). Interestingly, HDAC6 inhibition has been reported to restore nerve conduction and motor capacity in glycyl-tRNA synthetase (GARS)-mutated murine neuroblastoma cells, a model for CMT Type 2D (Benoy et al., 2018). Thus, if HDAC inhibitors succeed in showing a therapeutic effect in CMT2D, it would be interesting to further study if calcitriol could potentialize therapeutic effects of HDAC inhibitors in CMT2D diseases. To our knowledge, no study has been conducted yet on the impact of vitamin D3 on the progression of CMT disease. This could represent a new field of therapeutic research in CMT disease.

### A New Field of Research

Several questions should be raised to clearly assess the role of calcitriol in the peripheral nervous system. Does calcitriol have an impact on neuronal differentiation (and on axonal trophicity), or does calcitriol act more on Schwann cells myelination, or does calcitriol improve cellular communications between axons and Schwann cells thus improving myelination and nerve conduction velocities? This would imply a cellular, an animal and a human level of experiments.

For instance, at cellular level, neuronal differentiation comparing standard to calcitriol-supplemented cell cultures, may help assess if calcitriol induces or speeds-up neuronal differentiation. This could be performed on cell lines such as SH-SY5Y or on induced pluripotent stem cells (iPSc). Several techniques such as qRT-PCR, Western-blot and immunostaining on PGP9.5, islet, tuj1, HB9 expression, which are markers of neuronal differentiation, could assess an additive or a synergistic effect of vitamin D3 and other fat-soluble vitamins. Moreover, as micro-glial cells can synthetize calcitriol, 3D-cell culture including neuronal and glial cells could be relevant to study micro-environmental regulation of calcitriol synthesis.

At animal level, experiments could also be led on mice or rats with sciatic nerves injuries, physically or chemically induced, to determine if calcitriol has a positive impact on recovery. Numerous animal models exist for both acquired (toxic, diabetic, crush) and hereditary neuropathies (Sereda et al., 1996). Calcitriol would be administered orally or by a local long term delivery mean as done for curcumin for instance (Caillaud et al., 2018). Effects of calcitriol supplementation could be assessed by functional (skillful walking, grip strength, rotarod), histological (g-ratio) and electrophysiological (NCVs) tests. In these conditions, it would be important to check if calcitriol plays a role in the remyelination process and has synergistic effects with another factor as previously reported.

At a human level, as vitamin D3 is frequently given to elders, a prospective interventional study should be planned to monitor the incidence of peripheral neuropathies. Alternatively, as patients receiving a chemotherapy frequently develop neuropathies, a prospective interventional study could be envisioned to prevent those to occur, provided positive results to investigate in animal models.

## Conclusion

**FIGURE 3:**
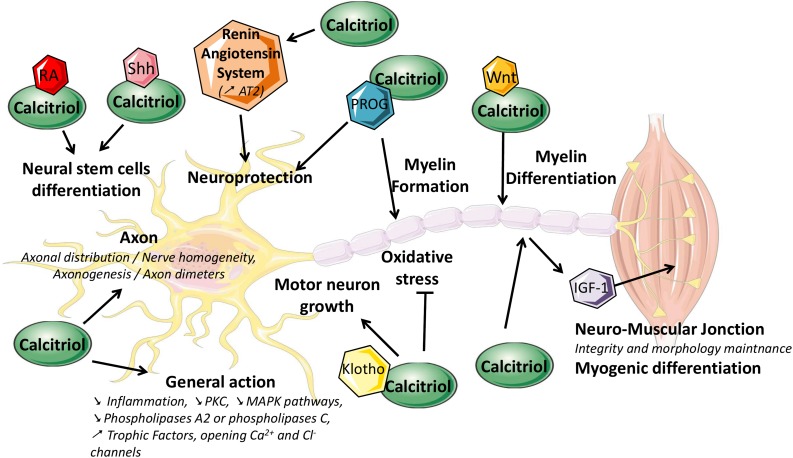
Schematic representation of the putative roles of calcitriol in the peripheral nervous system. IGF-1, insulin-like growth factor-1; MAPK, mitogen-activated protein kinase; PKC, protein kinase C; PROG, progesterone; RA, retinoic acid; Shh, Sonic hedgehog.

Basic science data suggest that current knowledge of calcitriol may still be incomplete and that it may play a more important role in peripheral nerve trophicity than previously thought (Figure 3). Several preliminary clinical studies tend to show that calcitriol, indeed, plays such a role. Future molecular and cellular studies may show calcitriol supplementation to be a beneficial means to positively influence peripheral nervous system homeostasis by regulating several processes, such as myelin genesis and axonal maintenance.

## Author Contributions

PAF and FP wrote a large proportion of the manuscript and searched for references. FM wrote some parts of the manuscript and inserted the references. ASL and CD proofread the sections concerning gene regulation and pharmacology. LM, as a neurologist, proofread the clinical part of the manuscript. FF wrote some parts of the manuscript and modified the general structure to make it more readable. FS initiated the work on vitamin D and proofread the manuscript several times.

## Conflict of Interest Statement

The authors declare that the research was conducted in the absence of any commercial or financial relationships that could be construed as a potential conflict of interest.

## Footnotes

1 www.clinicaltrials.gov

2 www.clinicaltrialsregister.eu
